# Engaging anaesthesia professionals as co-faculty in stroke thrombectomy simulation training: associations with clinical care and patient outcomes

**DOI:** 10.1186/s41077-025-00383-x

**Published:** 2025-10-24

**Authors:** Caroline Guldberg Fugelli, Hege Langli Ersdal, Soffien Ajmi, Jan Terje Kvaløy, Lars Fjetland, Cecilie Grøtteland, Snorre Eikeland, Victoria Brazil, Martin Wilhelm Kurz

**Affiliations:** 1 Department of Anaesthesia, Stavanger University Hospital, Stavanger, Norway; 2 Faculty of Health Sciences, University of Stavanger, Stavanger, Norway; 3 Department of Simulation-Based Learning, Stavanger University Hospital, Stavanger, Norway; 4 Department of Neurology, Stavanger University Hospital, Stavanger, Norway; 5 Department of Mathematics and Physics, University of Stavanger, Stavanger, Norway; 6 Department of Research, Stavanger University Hospital, Stavanger, Norway; 7 Stavanger Medical Imaging Laboratory (SMIL), Department of Radiology, Stavanger University Hospital, Stavanger, Norway; 8 Department of Clinical Medicine, University of Bergen, Bergen, Norway; 9 Business Intelligence Unit (Department of Analyses), Stavanger University Hospital, Stavanger, Norway; 10 Faculty of Health Sciences and Medicine, Bond University, Gold Coast, Australia

**Keywords:** Stroke, Ischaemic stroke, Thrombectomy, Simulation training, Anaesthesia, Translational simulation, Patient care team, Treatment outcome, Interprofessional education

## Abstract

**Background:**

Simulation-based training for endovascular thrombectomy (EVT) may improve care for patients with acute ischaemic stroke. This study investigated whether engaging anaesthesia professionals as co-faculty in an established in situ simulation programme influenced EVT care and patient outcomes.

**Methods:**

This single centre pre-post interventional study (2017-2023) evaluated the impact of revising an EVT simulation training programme by engaging anaesthesia professionals in its design, delivery, and debriefing. Outcomes were measured by changes in: (1) anaesthetic management (relative and absolute time systolic blood pressure was outside protocol thresholds, hypoxic time, and protocol adherence), (2) workflow process (time metrics, successful revascularisation rates, and intraprocedural complications), and (3) patient outcomes (intracerebral haemorrhage, National Institute of Health Stroke Scale scores and modified Rankin Scale scores).

**Results:**

A total of 275 stroke patients were treated with EVT during the study period (189 pre- and 86 postintervention). Anaesthetic management improved significantly in the postintervention group, with a decrease in the proportion of time that systolic blood pressure remained outside thresholds (37.0% to 27.7%, *p* = 0.02), increased compliance with recommended anaesthetics (27.5% to 100.0%, *p* < 0.001), and a reduction in hypoxia (5 to 0 min, *p* < 0.001). Time from suite arrival to groin puncture increased from 15 to 20 min in the postintervention group (*p* = 0.003). No significant differences were observed between the groups in the remaining workflow time metrics, reperfusion rates, or procedural complications. The proportion of patients with an excellent outcome (modified Rankin Scale 0–1) improved significantly from 23.4% to 42.6% in the postintervention group (*p* = 0.01).

**Conclusions:**

The engagement of anaesthesia professionals in the EVT simulation training faculty was associated with improved EVT anaesthesia care and improved patient outcomes. The multidisciplinary nature of the EVT team should be reflected in faculty composition for EVT simulation training.

**Supplementary Information:**

The online version contains supplementary material available at 10.1186/s41077-025-00383-x.

## Introduction

Endovascular thrombectomy (EVT) simulation training can enhance clinical care for acute large vessel occlusion stroke patients, but these outcomes are not uniformly achieved [1]. The design and delivery of simulation training are variable, as are the contexts in which it is delivered. Without adequate stakeholder engagement in simulation design, training may be ineffective and offer poor return on investment. We aimed to test whether engaging anaesthesia professionals as co-faculty in an established in situ simulation training programme improved EVT clinical care and patient outcomes.

Stroke remains a leading cause of morbidity and mortality worldwide, imposing a significant socioeconomic burden [2]. In acute ischaemic stroke, intravenous thrombolysis (IVT) is often the initial treatment for eligible patients. Depending on timing, stroke characteristics, and imaging findings, IVT may be combined with or replaced by EVT, a mechanical procedure to remove the clot. EVT for large vessel occlusion ischaemic stroke has been a major advancement in improving patient outcomes [3]. Unlike IVT, which aims to dissolve the clot pharmacologically and involves fewer procedural steps, EVT is a technically complex catheter-based intervention requiring multidisciplinary coordination, including interventional radiology and anaesthesia personnel. EVT success depends on multiple factors, including endovascular techniques, anaesthetic management, patient physiology, and team efficiency [4–7]. Timely treatment and effective multidisciplinary collaboration are particularly crucial for optimal results [8, 9].

Simulation-based training for EVT has shown promise in reducing treatment times and enhancing the perceived teamwork climate [10, 11]. However, while IVT simulation has demonstrated improvements in treatment times and patient outcomes, the impact of EVT simulation on team performance and outcomes remains underexplored. Building on the success of our IVT simulation programme, we initially extended this model to EVT in 2017 [1]. While this intervention significantly reduced workflow times, it did not translate into a corresponding significant improvement in patient outcomes [12]. We identified suboptimal anaesthetic management during EVT, as anaesthesia personnel were not engaged in the EVT simulation training faculty, as a potential contributing factor.

This discrepancy highlights the challenge of replicating successful interventions across different healthcare settings, a concept explored within the framework of translational simulation. Translational simulation connects simulation training with real-world health service priorities and patient outcomes [13]. It promotes a ‘context logic’ approach, embracing complexity, wherein simulation training not only replicates clinical task interactions but also considers work environments, team dynamics, and organisational culture that influence performance [14]. Given that traditional simulation models have not consistently translated into improved patient outcomes, adopting a translational simulation approach allows for targeted modifications that align with real-world clinical priorities. Through this lens, the effective design and implementation of simulation-based improvement requires the engagement of all subgroups within a multidisciplinary team. For EVT simulation training, anaesthesia is one important subgroup.

Anaesthesia plays a critical role in EVT by maintaining patient stability and optimising conditions for thrombus extraction [15]. Maintaining adequate systolic blood pressure (SBP) is particularly crucial for minimising ischaemic cerebral damage [16]. EVT anaesthesia, classified as a nonoperating room anaesthesia (NORA) procedure, is often complicated by challenges such as limited space, unfamiliarity among team members, and high-pressure situations [17–19]. These factors underscore the importance of integrating anaesthesia professionals into the design of EVT simulation training to foster shared understanding and improve overall team efficiency.

This study evaluated whether engaging anaesthesia professionals in the design, delivery, and debriefing of a multidisciplinary EVT simulation training programme was associated with changes in clinical anaesthetic management, workflow processes, and patient outcomes.

## Methods

### Study design

This single-centre, prospective pre-post intervention study was part of a mixed-methods project exploring the impact of EVT simulation training on clinical outcomes. It was designed as an exploratory quantitative follow-up study, informed by a prior qualitative needs assessment study [20].The Regional Committee for Medical and Health Research approved the study (2018/1895). All procedures were conducted in accordance with the Declaration of Helsinki and adhered to national and institutional standards. The study follows the SQUIRE (Standards for Quality Improvement Reporting Excellence Guidelines) and reporting guidelines for healthcare simulation research [21, 22].

### Study context

The study was conducted at a Norwegian stroke centre serving a population of 380,000. The centre performs approximately 50–60 EVT procedures annually, supported by a multidisciplinary EVT team available 24/7. The team includes two emergency department (ED) nurses, two radiographers, one or two interventional radiologists, a nurse anaesthetist, an anaesthetist, a neurology registrar, and a consultant neurologist. The interventionists were generalists, and there was no specialised neuroanaesthesia team.

In 2017, a quality improvement (QI) team of neurology and radiology professionals implemented an in situ EVT simulation training programme aimed at improving acute stroke care. This was integrated with an existing IVT simulation training programme, enabling each session to simulate the entire in-hospital acute stroke care pathway, from ED arrival to reperfusion. Weekly in situ training sessions were conducted once or twice annually and were grouped into clusters over four-month periods. Although anaesthesia personnel regularly participated in simulations as part of the clinical team, they were not involved in the design, delivery, or debriefing of the training. Notably, one anaesthetist had been responsible for developing and maintaining the clinical anaesthesia protocol for EVT since 2017, but had not been engaged in simulation training for the procedure.

Data collected in 2021 to evaluate the effect of the EVT simulation training programme revealed significant time savings in procedural time to reperfusion, but neither anaesthetic management nor patient outcomes improved [12]. A subsequent qualitative study, involving focus group interviews with EVT team members from all relevant disciplines, identified the need for a revised EVT protocol and an increased focus on the EVT procedure within the broader stroke simulation training programme [20]. The study findings also emphasised the importance of enhanced training for anaesthetic management.

### EVT workflow

The preliminary decision regarding EVT treatment is made in the CT scanner, after imaging is completed, triggering a call to notify the entire EVT team. The neurology registrar, accompanied by the ED nurses, escorts the patient to the angio suite. The consultant neurologist and interventional radiologist, located in a consultation room, review the imaging and decide whether to proceed with EVT. Upon patient arrival at the angio suite, the ED nurses and anaesthesia team (one nurse anaesthetist and one anaesthetist) prepare the patient for anaesthesia, whereas two radiographers prepare for the interventional procedure. Once the consultant neurologist and interventional radiologist have made the final decision to proceed with EVT, they join the rest of the team in the angio suite. The procedure then progresses with anaesthesia induction, groin puncture, and initiation of the radiology intervention. The neurology registrar leads the team until the final decision to proceed with EVT is confirmed, at which point the consultant neurologist assumes the role of team leader. A “Safe Thrombectomy” checklist, covering key safety and procedural elements, should be used by the team leader at three key stages: upon patient arrival in the angio suite, immediately before groin puncture, and at the end of the procedure.

### Intervention

The intervention was built directly upon modifications identified in our previous research, which have been published separately [12, 20], and had three components (Table 1):

1. Change in simulation faculty composition (engaging anaesthesia professionals)
2. Revision to the clinical EVT protocol (switching to general anaesthesia)
3. Enhancements to EVT simulation training

**Table 1:** Changes to EVT protocol, suite, and training following engagement of anaesthesia professionals in simulation faculty

EVT protocol revision	• Implementing an improved “safe thrombectomy” checklist to enhance team members’ shared understanding of critical steps and responsibilities • Clearly defining task distribution among team members upon patient arrival in the angio suite • Adopting general anaesthesia as the primary choice of anaesthesia • Prefilled propofol and remifentanil syringes for infusion pumps
EVT suite revision	• Installing a large monitor in the angio suite to display patients’ vital signs prominently, ensuring visibility for the entire EVT team
EVT simulation training revision	• Providing independent EVT training from the overall stroke simulation training to ensure sufficient time for focused learning goals • Implementing a patient monitor simulator for more realistic use of patient monitoring equipment • Replacing one of the manikin's arms with a "wet" arm capable of fluid infusion • Ensuring essential technical task performance within a realistic timeframe (inserting and securing peripheral intravenous catheters, preparing and administering drugs, preparing and connecting infusion pumps, inserting urinary catheters, sterile draping, etc.) • Implementing common anaesthetic challenges encountered in the clinical setting (haemodynamic and respiratory instability, critical moments during procedures, etc.) • Focusing on the importance of the role of team leaders in facilitating a shared understanding • Ensuring the presence of clinical EVT team members • Engaging a simulation expert as an observer in the EVT simulations and a contributor to the debrief • Implementing a revised presimulation e-learning programme • Providing regular interprofessional education • Developing an educational video to explain how anaesthesia works to the non-anaesthesia members of the EVT team

*Abbreviations*: *EVT* Endovascular thrombectomy

In November 2021, one anaesthetist and two nurse anaesthetists were engaged as co-faculty in the EVT simulation training programme. The anaesthetist, who had overseen the clinical anaesthesia protocol since 2017, was among those newly engaged. This marked a shift, expanding the simulation faculty to include anaesthesia professionals for the first time. All faculty members were active clinicians regularly involved in EVT procedures, and most held clinical leadership roles within their specialties. This integration supported a bidirectional exchange between simulation-based insights and clinical practice, contributing to collaborative revisions to both the EVT clinical protocol and the simulation training.

Revisions to the protocol were developed by the newly expanded faculty, based on findings from the initial EVT simulation training. These findings included a lack of improvement in haemodynamic management and considerable variation in the anaesthetic agents used, issues we collectively defined as suboptimal anaesthetic management [12]. Additionally, qualitative findings highlighted EVT team frustration regarding the choice between sedation and general anaesthesia [20]. In response, the protocol was revised to adopt general anaesthesia with intubation as standard practice (see Additional file 1: Supplementary Table S1) for the revised anaesthesia protocol). The existing “Safe Thrombectomy” checklist was also updated to clarify team roles, strengthen leadership, and promote a shared understanding among team members. To address identified workflow challenges and promote standardisation, several ergonomic adjustments were made in the clinical environment. Prefilled propofol and remifentanil syringes were made available for infusion pump use to reduce preparation time and facilitate smoother induction of anaesthesia. A large wall-mounted monitor was installed in the angio suite to prominently display patient vital signs, aiming to enhance shared situational awareness across the team.

A revised EVT simulation training was then developed, with the anaesthesia co-faculty actively engaged in its design, delivery, and debriefing. Our description of the training programme that follows is structured according to the reporting guidelines of Cheng et al. [22]. All faculty members were certified as Level 1 EuSim simulation facilitators [23]. Between November 2021 and June 2022, 18 in situ simulation sessions were conducted to train all on-call anaesthetists, followed by 5 to 6 simulation sessions every 6 months.

Several enhancements were introduced to the simulation training, some at the outset and others developed iteratively in response to emerging insights from simulation and clinical practice. The first involved separating EVT simulation sessions from the broader stroke simulation training to allow focused procedural training. The second focused on participation. Recognising that experienced anaesthesia personnel had previously been underrepresented in simulation training, the programme aimed to include all anaesthetists who were part of the on-call EVT team. To achieve this,18 in situ simulation sessions were conducted between November 2021 and June 2022. This was followed by 5 to 6 simulation sessions every 6 months, consistently involving active members of the EVT team. Building on the previous EVT simulation training programme, these sessions reiterated three key learning objectives: knowledge and compliance with the revised protocol, efficient task distribution, and overall team effectiveness. The participants were prebriefed via a revised e-learning programme aligned with the new protocol, supplemented by an email reminder one week prior to each session. These materials included instructions on how to orient themselves to the simulator and learning objectives, which were reinforced orally by the faculty immediately before each training session. In addition to the e-learning, regular interdisciplinary education sessions were conducted, and an educational video was developed to explain anaesthetic work to non-anaesthesia team members, supporting shared understanding and role clarity across professions. The simulations began with an activated EVT call, as in clinical practice. ED nurses and a neurology registrar transferred a standardised patient from the CT scanner to the angio suite. The standardised patient was replaced by a manikin upon arrival, where the remaining EVT members awaited. The participants assumed their usual clinical roles. The third area of enhancement focused on simulation modalities to better reflect clinical task interactions. A high-fidelity manikin (Sim Man Vascular, Laerdal Medical, Stavanger, Norway) was modified with a “wet” arm for medication and fluid administration. A patient monitor simulator (FLUKE ProSim 8 Vital Signs and ECG Patient Simulator, Fluke Biomedical, Washington, USA) was introduced to replicate clinical patient monitoring, enabling use of the same monitors as used in daily practice. The virtual interventional radiology simulator (Mentice VIST® G5 simulator, Mentice AB, Gothenburg, Sweden) remained unchanged. Clinical equipment was used throughout, except for fluids for radiological tasks to prevent manikin damage. The final set of enhancements addressed realistic “everyday challenges”, including postinduction hypotension, allergic reactions, arrhythmia from wire placement in critical areas, and dislodged intravenous infusions. These challenges reflected issues previously identified in our research as common sources of stress and communication breakdown during clinical teamwork, yet had not been addressed in prior simulation training [20]. Their inclusion aimed to improve interprofessional communication, highlight task dependencies between anaesthesia and radiology, and emphasise the importance of present and clear team leadership. The scenarios were not prebriefed to the participants. Each 30-min simulation session was followed by a 30-min debriefing session. Anaesthesia and neurology faculty facilitated the sessions, with radiographers and a nurse anaesthetist managing the technical aspects. A neurologist led the debriefings, co-facilitated by an anaesthetist.

A simulation expert was engaged as an independent observer during the simulation sessions. This was prompted by faculty reflections that prior debriefings, primarily led by neurologists and radiologists, might have been shaped by professional perspectives, limiting interdisciplinary learning. The simulation expert’s role was to observe the full simulation sessions, with particular focus on the debriefings, and provide feedback on alignment with established debriefing frameworks and best practices. When specific issues arose during the debrief, such as areas identified for improvement in the simulation training or clinical practice, the simulation expert documented these observations in a written summary. These summaries were shared with all faculty members to support the ongoing refinement of the training programme.

### Data collection and variables

#### Patients

All patients treated with EVT at our center from November 2017 to June 2023 were registered. Patients were excluded from analyses of anaesthetic management if haemodynamic data or procedural time within anaesthesia care were missing in the anaesthesia electronic medical records system (Picis Anaesthesia Manager, Picis Clinical Solutions, Inc., Wakefield, Boston, MA, USA) or if the procedure exceeded 240 min. Patients were excluded from analyses of workflow process and patient outcomes if they had a pre-EVT score of 2 or higher on the modified Rankin Scale (mRS), underwent multiple EVT procedures during the same hospital stay, died of cancer within 3 months, had procedures exceeding 240 min, or were treated according to DAWN protocol criteria involving clinical-imaging mismatch assessed by advanced imaging [24]. The latter were excluded as a distinct subgroup due to their extended treatment time windows and different care pathways. The 240-min time limit was chosen to avoid extreme outliers.

#### Outcomes

The primary outcome was haemodynamic management, measured as the proportion of time that SBP remained outside defined protocol thresholds of 140–180 mmHg during anaesthesia care. This metric reflects the established importance of maintaining SBP within target ranges during EVT, and using a relative time accounted for variations in procedure duration [25]. The secondary outcomes, detailed below, included (1) changes in anaesthetic management, (2) workflow process, and (3) patient outcomes.

1. Anaesthetic management
  For haemodynamic management, data were also collected on the absolute duration (minutes) that SBP remained outside defined protocol thresholds during anaesthesia care. In addition, we collected data on the absolute duration (minutes) of hypoxia, defined as values outside the recommended protocol thresholds, and adherence to the recommended anaesthetic agents as outlined in the EVT anaesthesia protocol (see Additional file 1 (Supplementary Table S1) for protocol details and threshold definitions).
2. Workflow process
  We collected data on time metrics, the successful revascularisation (TICI score 2b-3) rate, and the frequency of intraprocedural complications.
3. Patient outcomes
  Data were collected on the incidence of intracerebral haemorrhage (ICH), defined according to the European Cooperative Acute Stroke Study II (ECASS II) classification. Symptomatic ICH was defined as any ICH associated with a neurological deterioration of ≥ 4 points on the NIHSS within 24 h. Data were also collected on the National Institute of Health Stroke Scale (NIHSS) score, and the mRS score. The mRS score was assessed 90 days after stroke onset and dichotomised into “excellent” (mRS 0–1), “good” (mRS 0–2) and “worst” (mRS 5–6), in accordance with previous studies [1, 3, 26].

### Statistical analysis

Sample size calculations were based on the proportion of time that SBP remained outside defined protocol thresholds during anaesthesia care. A clinically significant difference of 13 percentage points was selected, which is consistent with our previous study on EVT simulation training [12]. Assuming a standard deviation (SD) of 23 percentage points, a minimum sample size of 47 patients was required to achieve a power of 0.8, with a two-sided t-test, and a significance level of *p* < 0.05 was used.

The quantitative outcome measures included both continuous variables (time, NIHSS score) and categorical variables (e.g., adherence to the anaesthesia protocol, rate of successful reperfusion, mRS score dichotomised). Continuous variables were non-Gaussian and are presented as medians with first and third quartiles. Group differences were assessed using the Mann–Whitney *U* test. Categorical variables are reported as absolute numbers and frequencies, with differences analysed using the Pearson chi-square test. To assess whether the change in clinical outcome could be explained by changes over time in clinical factors known to influence patient outcome, we performed a multivariable logistic regression analysis for the patient outcome of mRS 0–1, adjusting for relevant clinical factors. Statistical significance was defined as a 2-tailed *p*-value < 0.05. Analyses were conducted using SPSS Statistics version 26 (IBM Cooperation, Armonk, NY, USA) and R version 4.3.3 [27].

## Results

The results are presented in the following categories: (1) Patient characteristics, (2) Anaesthetic management, (3) Workflow process and (4) Patient outcomes.

### Patient characteristics

**Fig. 1:**
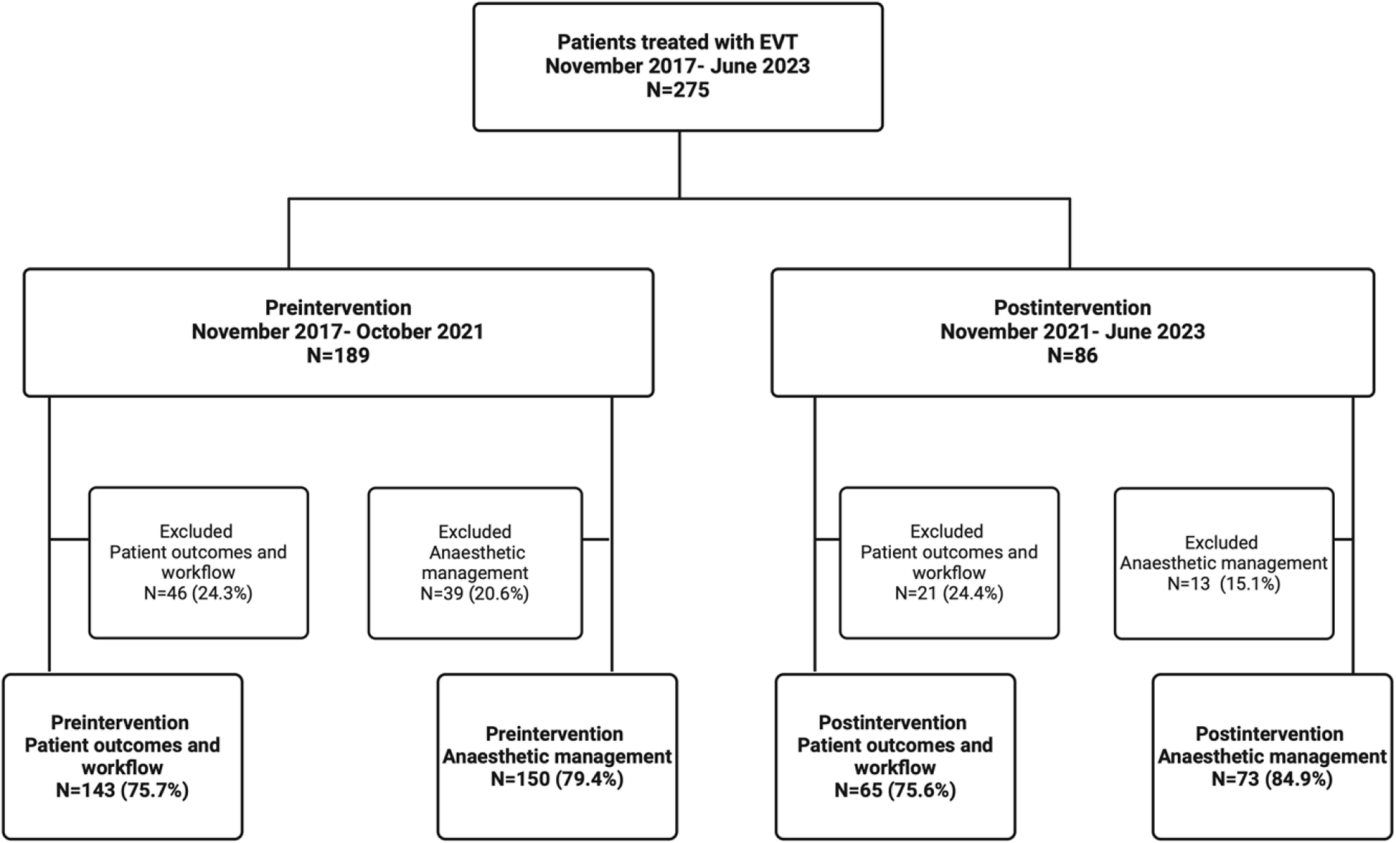
Overview of patients included. Flowchart illustrating the number of patients screened, excluded, and included in each analysis cohort. Abbreviations: EVT: endovascular thrombectomy

Between November 2017 and June 2023, a total of 275 stroke patients underwent EVT procedures at our hospital. Of these, 189 patients were treated in the preintervention period and 86 patients were treated in the postintervention period. For the evaluation of anaesthetic management, 52 patients were excluded. For the evaluation of the workflow process and patient outcomes, 67 patients were excluded (Fig. 1). Detailed reasons for patient exclusion are presented in Additional file 1 (Supplementary Table S2). No significant differences were found between the pre- and postintervention groups in terms of age, sex, interhospital transport/ inhospital stroke onset or comorbidities (Table 2). However, patients in the postintervention group presented with a significantly lower initial NIHSS score (16.0 preintervention versus 13.0 postintervention, *p* = 0.01) and a significantly lower rate of bridging thrombolysis (65.7% preintervention versus 50.8% postintervention, *p* = 0.048).

**Table 2:** Comparison of patient characteristics before and after the intervention

Anaesthetic management cohort	Preintervention (*n* = 150)	Postintervention (*n* = 73)	*P* Value
Age (years)	74.5 (63.8–82.3)	76.0 (67.0–82.5)	0.41
Female	64 (42.7)	40 (54.8)	0.09
Interhospital transport/inhospital stroke onset	53 (35.3)	24 (34.8)	0.94
Comorbid conditions			
Atrial fibrillation	68 (45.6)	29 (39.7)	0.40
Diabetes mellitus	28 (18.8)	16 (21.9)	0.58
Hypertension	82 (55.0)	44 (60.3)	0.46
Previous myocardial infarction	24 (16.1)	15 (20.5)	0.41
Previous cerebral infarction	18 (12.1)	11 (15.1)	0.54
Clinical variables			
Initial NIHSS	16.0 (10.5–21.5)	13.0 (8.0–19.0)	0.02
Initial mRS	0.0 (0.0–0.0)	0.0 (0.0–0.0)	0.64
Initial ASPECT score	8.0 (6.0–10.0)	9.0 (7.0–10.0)	0.17
Bridging thrombolysis	90 (60.0)	37 (55.2)	0.51
Anterior circulation occlusion site	134 (89.3)	66 (90.4)	0.80
Invasive arterial blood pressure monitoring	148 (98.7)	72 (98.6)	0.98
Workflow process and patient outcomes cohort	Preintervention (*n* = 143)	Postintervention (*n* = 65)	*P* Value
Age (years)	74.0 (62.0–81.0)	74.0 (67.0–80.0)	0.69
Female	61 (42.7)	34 (52.3)	0.20
Interhospital transport/inhospital stroke onset	47 (32.9)	20 (32.8)	0.99
Comorbid conditions			
Atrial fibrillation	61 (42.7)	24 (36.9)	0.44
Diabetes mellitus	22 (15.4)	11 (16.9)	0.78
Hypertension	79 (55.2)	38 (58.5)	0.67
Previous myocardial infarction	24 (16.8)	11 (16.9)	0.98
Previous cerebral infarction	17 (11.9)	7 (10.8)	0.82
Clinical variables			
Initial NIHSS	16.0 (11.0–21.0)	13.0 (7.0–18.0)	0.01
Initial mRS	0.0 (0.0–0.0)	0.0 (0.0–0.0)	0.78
Initial ASPECT score	8.0 (6.0–10.0)	9.0 (6.0–10.0)	0.11
Bridging thrombolysis	94 (65.7)	30 (50.8)	0.048
Anterior circulation occlusion site	132 (92.3)	58 (89.2)	0.47
Invasive arterial blood pressure monitoring	137 (97.9)	64 (98.5)	0.77

*Abbreviations*: *ASPECT* Alberta stroke program early CT score, *mRS* Modified Rankin scale, *NIHSS* National institutes of health stroke scale/score

Numbers are reported as numbers (percentages) for categorical variables and medians (quartiles) for continuous variables

Variables with varying sample sizes due to data availability are listed in Additional file 1 (Supplementary Table S3)

### Anaesthetic management

The primary outcome measure (haemodynamic management) improved significantly, with the proportion of time that SBP remained outside the recommended thresholds decreasing from 37.0 to 27.7% (*p* = 0.02) (Table 3). Compliance with recommended anaesthetic protocols also improved significantly from 27.5% preintervention to 100% postintervention (*p* < 0.001).

**Table 3:** Comparison of anaesthetic management before and after the intervention

	Preintervention (*n* = 150)	Postintervention (*n* = 73)	*P* Value
Primary sedation	115 (76.7)	0 (0.0)	< 0.001
General anaesthesia (incl. conversions)	52 (34.9)	72 (98.6)^a^	< 0.001
Compliance with recommended anaesthetics, sedation	49 (43.4)		
Compliance with recommended anaesthetics, general anaesthesia	14 (27.5)	72 (100.0)	< 0.001
Conversion from sedation to general anaesthesia	18 (12.0)		
Procedure time within anaesthesia care (min)	82.5 (57.8–111.0)	88.0 (59.5–120.0)	0.62
SBP outside threshold (min)	28.5 (14.8–43.0)	23.0 (11.5–39.5)	0.13
SBP outside threshold (% of procedure time)	37.0 (20.0–54.7)	27.7 (14.5–41.4)	0.02
Hypotension (% of procedure time)	14.6 (5.8–41.2)	11.8 (5.2–23.4)	0.18
Hypertension (% of procedure time)	4.6 (3.4–20.3)	7.3 (2.5–17.6)	0.30
Hypoxia (min)	5.0 (1.0–18.3)	0.0 (0.0–2.0)	< 0.001

*Abbreviations*: *SBP* Systolic blood pressure

Numbers are reported as numbers (percentages) for categorical variables and medians (quartiles) for continuous variables

^a^One patient in the postintervention group received no sedation or general anaesthesia due to comorbidities

### Workflow process

There were no significant changes in onset-to-angio suite arrival times, onset-to-reperfusion times, or other major workflow variables (Table 4). However, the time from angio suite arrival to groin puncture increased significantly (15.0 min preintervention vs. 20.0 min postintervention, *p* = 0.003), though groin puncture to reperfusion times remained similar (53.0 min preintervention vs. 55.0 min postintervention, *p* = 0.99).

**Table 4:** Comparison of the EVT workflow and patient outcomes before and after the intervention

	Preintervention (*n* = 143)	Postintervention (*n* = 65)	*P* Value
Workflow Process			
Onset- Angio suite arrival	119.5 (79.3–207.3)	122.0 (72.5–216.5)	0.75
Onset-reperfusion	210.0 (163.0–295.3)	191.5 (141.8–260.8)	0.19
Door- Angio suite arrival	35.0 (25.5–50.0)	33.5 (29.0–61.8)	0.33
Door-reperfusion	112.5 (89.5–152.8)	129.0 (100.0–157.3)	0.21
Angio suite arrival-groin puncture	15.0 (10.0–21.8)	20.0 (13.0–28.0)	0.003
Groin puncture-reperfusion	53.0 (37.0–79.0)	55.0 (38.0–82.0)	0.99
TICI score 2b-3	124 (86.7)	57 (87.7)	0.85
Interventional complications	27 (19.6)	7 (10.9)	0.13
Patient Outcomes			
ICH	5 (3.5)	3 (4.7)	0.68
mRS 0–1	33 (23.4)	26 (42.6)	0.01
mRS 0–2	70 (49.6)	38 (62.3)	0.10
mRS 5–6	19 (13.5)	10 (16.4)	0.59
NIHSS post-EVT	9.0 (3.0–17.0)	6.0 (2.0–11.5)	0.03
NIHSS difference at 24 h	4.0 (0.0–11.3)	5.0 (0.0–10.0)	0.67
NIHSS at discharge	6.0 (1.0–11.0)	2.5 (0.0–8.3)	0.04
NIHSS difference at discharge	9.0 (3.0–14.0)	7.0 (2.8–12.3)	0.20

*Abbreviations*: *EVT* Endovascular thrombectomy, *ICH* Intracerebral haemorrhage, *mRS* Modified Rankin scale, *NIHSS* National institutes of health stroke scale/score, *TICI* Thrombolysis in cerebral infarction

Time variables are reported as minutes. Numbers are reported as numbers (percentages) for categorical variables and medians (quartiles) for continuous variables

Variables with varying sample sizes due to data availability are listed in Additional file 1: Supplementary Table S3

### Patient outcomes

The proportion of patients with an excellent clinical outcome at 90 days (mRS 0–1) increased significantly from 23.4% to 42.6% (*p* = 0.006) postintervention (Table 4). This difference remained significant after adjusting for successful reperfusion, initial NIHSS and bridging thrombolysis [OR 2.60 (CI 1.26–5.35), *p* = 0.01]. No significant changes were observed in mRS 0–2 or mRS 5–6. NIHSS scores significantly improved at 24 h post-EVT (median 9.0 preintervention vs. 6.0 postintervention, *p* = 0.03) and at discharge (median 6.0 vs. 2.5, *p* = 0.04), though they lost significance when compared to the initial NIHSS at stroke diagnosis (Table 4). ICH occurred in 5 of 143 patients (3.5%) in the preintervention group and in 3 of 65 patients (4.7%) in the postintervention group (*p* = 0.68); none of the cases were classified as symptomatic.

## Discussion

Our study suggests that engaging anaesthesia professionals in an EVT simulation training programme is associated with more stable anaesthetic management, enhanced team functioning, and improved patient outcomes. In this discussion, we offer reflections on teamwork in EVT for stroke care and the role and design of simulation in improvement for this context.

Delivering EVT simulation training requires a multidisciplinary focus that extends beyond time efficiency, recognising the complexity of stroke patient care and the critical role of teamwork. Teamwork, here defined as the interrelated attitudes, behaviours, and cognitions required to perform the interdependent actions of the team, is recognised as the most critical factor contributing to team performance [28]. In our study, prior findings of suboptimal anaesthetic management suggested that teamwork was not optimal and that interventions targeting team dynamics within EVT had the potential to improve overall team performance. Engaging anaesthesia staff as co-faculty in the simulation training programme represented a deliberate intervention aimed at addressing this gap. This faculty integration did not function as an isolated intervention, but acted within system complexity, offering important perspectives that guided revisions to both the training and the clinical procedure through an iterative, collaborative process.

Our results underscore the value of simulation-based improvement embracing the broader clinical system, a core principle of the translational simulation framework [29]. A key feature of our model is that simulation is not externally imposed or confined to an educational silo. Instead, it is embedded in a real-world context where the simulation faculty are also frontline clinicians and clinical leaders. This integration enables a unique integration of simulation, clinical insights, and quality improvement. Attempting to isolate the impact of individual components may overlook the value of this collective, iterative approach. In our study, simulation serves both *exploratory* and interventional purposes. The design of our interventions aligns with this context-sensitive, iterative, and outcome-driven translational simulation approach in multiple respects. First, the framework highlights the importance of defining the problem to be addressed. In our case, suboptimal anaesthetic management was identified as a priority for improving team performance and stroke care. Second, it underscores the value of engaging relevant stakeholders to effectively design simulation-based interventions. In our study, the inclusion of anaesthesia professionals as co-faculty informed several key modifications, including environmental interventions (the installation of a wall-mounted patient monitor in the angio suite to support shared situational awareness) and simulation modality modification selection (replacing the manikin’s “dry” arm with a “wet” arm to enable fluid administration). Thus, the extension of the IVT simulation model into the EVT setting could better reflect differences in the clinical environment, task complexity, and team composition. Third, the ‘context logic’ approach within translational simulation encourages exploration and shaping of the work environment and the people within it [14]. Our revised EVT-specific simulation training supported the setting’s unique teamwork challenges. It targeted relevant factors across the full context of team performance, including organisational, environmental, and workflow processes. These changes were not implemented all at once, but rather evolved over time in response to continuous simulation-based and clinical insights, a characteristic feature of complex system improvement. Finally, translational simulation embraces implementation factors as powerfully as interventional factors. At the organisational level, we ensured that departmental leadership committed adequate resources and time for simulation training, allowing both novice and experienced EVT team members to participate.

The interventions aimed at improving workflow involved updating protocols and providing interprofessional education, focusing on critical teamwork factors such as role clarity, task allocation, and communication strategies [30]. These educational efforts were intended to foster shared understanding across professions and help bridge differences in clinical assumptions. The revised EVT protocol sought to reduce stressors commonly encountered in the NORA setting, such as unfamiliarity with the environment and communication style [18]. Standardising the anaesthetic approach to general anaesthesia was one such adjustment, aimed at reducing reported interprofessional frustrations and improving teamwork coordination. While general anaesthesia in the EVT setting is often associated with increased haemodynamic instability, our observations suggest the opposite [31, 32]. We believe this may reflect the benefits of tailoring simulation programmes and their interventions to a specific clinical context. Additionally, we sought to strengthen leadership practices by enhancing the procedural checklist to support team leaders in fostering shared mental models and guiding team performance more effectively.

This study has several limitations. First, the pre-post interventional design limits causal inference, particularly in the context of a complex, system-level intervention. However, engaging anaesthesia professionals as co-faculty represented a distinct and well-defined change, allowing for meaningful comparison with our institution’s prior simulation programme. Second, the fact that our simulation faculty were also active clinicians and clinical leaders in EVT care likely enhanced the relevance and impact of the training. However, this embedded model may not be easily replicable in other institutions where simulation is a more stand-alone educational activity, separate from clinical governance or quality improvement initiatives. In such contexts, fostering strong partnerships with clinical leaders and embedding structured feedback mechanisms may provide alternative pathways to achieve similar system-level impacts. Conversely, the involvement of a simulation expert as an independent observer may have helped balance the potential influence of profession-specific biases during debriefings. In settings where simulation faculty are predominantly clinicians, integrating simulation expertise can offer valuable methodological oversight and promote reflective, team-based learning. Third, patients in the postintervention group had significantly lower initial NIHSS scores, which may have contributed to improved outcomes. We adjusted for this in our regression analysis, but residual confounding cannot be fully excluded. Fourth, the lower rate of bridging thrombolysis in the postintervention group may have influenced results, although this could have biased findings toward the null rather than explaining the improvements observed [33]. Finally, our focus was on in-hospital EVT treatment, and we recognise that the broader stroke care pathway also plays an important role in patient outcomes and was beyond the scope of this investigation.

Our findings provide valuable insights for the development of EVT simulation training programmes. Given that EVT teams operate within complex healthcare systems, we argue that the translational simulation principle of addressing the broader clinical context should be central to simulation planning. A simulation training programme that is effective for one stage of the patient journey, such as acute stroke care, may not be effective for other stages of the patient journey, when new tasks and professional roles are introduced. As such, adaptations to simulation design should account for these changes and actively engage emerging stakeholders. This flexible, context-aware approach is essential for sustaining relevance and impact.

Our findings may have relevance beyond EVT, particularly for other NORA procedures within radiology, such as embolisation for trauma or postpartum haemorrhage and catheter-directed thrombolysis for pulmonary embolism. In these multidisciplinary settings, effective collaboration with anaesthesia professionals is critical for optimising patient outcomes, and their input should be actively solicited and integrated into simulation planning and delivery.

In conclusion, this study highlights the importance of designing simulation training programmes that reflect the multidisciplinary nature of EVT teams. Engaging anaesthesia professionals as co-faculty was associated with clinical changes, such as more stable anaesthetic management, alongside key protocol revisions, which coincided with better patient outcomes. These findings emphasise the value of inclusive, team-based simulation design in optimising care for complex procedures such as EVT and point to the potential of translational simulation to drive meaningful improvements in clinical practice.

## Supplementary Information

Additional file 1: Supplemental Table S1. Anaesthesia protocol for endovascular thrombectomy before and after intervention. Supplemental Table S2. Overview of excluded patients and reasons for exclusion. Supplemental Table S3. Sample size variations by variable due to data availability.

## Data Availability

The data that support the findings of this study are available upon request from the corresponding author. The data are not publicly available due to privacy or ethical restrictions.
