# Supplementary material for: Engaging anaesthesia professionals as co-faculty in stroke thrombectomy simulation training: associations with clinical care and patient outcomes
Source: Adv Simul (Lond). 2025 Oct 24;10:52. doi: 10.1186/s41077-025-00383-x (PMC12551271; doi:10.1186/s41077-025-00383-x)
Supplement: Supplementary file 1 — Additional file 1: Supplemental Table S1. Anaesthesia protocol for endovascular thrombectomy before and after intervention. Supplemental Table S2. Overview of excluded patients and reasons for exclusion. Supplemental Table S3. Sample size variations by variable due to data availability. [file 41077_2025_383_MOESM1_ESM.docx]

**Additional file 1**

**Supplemental Table 1.** Anaesthesia protocol for endovascular thrombectomy before and after intervention

|  | Preintervention (November 2017-October 2021) | Postintervention  (November 2021-June 2023) |
| --- | --- | --- |
| Primary anaesthetic choice | Conscious sedation;  Propofol infusion 1-2 mg/kg/h and fentanyl 25-50 μg bolus iv | General anaesthesia with intubation;  Propofol infusion 1-3 mg/kg/h and remifentanil infusion 0.05-0,1 μg/kg/min iv. |
| Secondary anaesthetic choice | General anaesthesia indicated when;   - Patients are unconscious or threatened airway - Patients have nausea and/or vomiting - Not cooperating with restless behaviour - Posterior occlusion   Sevoflurane inhalation and remifentanil infusion 0.05-0.1 μg/kg/min iv. | Conscious sedation indicated when;  Individual decision based on patient comorbidities. Discussion involving neurologist, interventional radiologist and anaesthetist. |
| Vital parameters thresholds | | |
| Systolic Blood Pressure | 140-180 mmHg | 140-180 mmHg |
| SpO2 | ≥93 %  (≥96% Nov 17-Nov 18) | ≥93 % |
| EtCO2 | <6 kPa | <6 kPa |
| Temperature | <38°C | <38°C |
| Blood glucose | <10 mmol/L | <10 mmol/L |
| Vasoactive treatment | Hypotension:   - Norepinephrine infusion iv 0.02-0.2 micrograms/kg/min - Ephedrine bolus 5-10 mg iv   Hypertension:   - Labetalol bolus 10-20 mg iv or continuous infusion when indicated | |
| End of procedure | Extubation as early as possible, preferably in the angio suite. Postoperative care in the stroke unit if successfully extubated. | |

**Supplemental Table 2.** Overview of excluded patients and reasons for exclusion

| **Cohort** | **Time period** | **Total EVT-treated patients (n)** | **Excluded patients (n, %)** | **Exclusion reasons** | **n (% of excluded patients)** |
| --- | --- | --- | --- | --- | --- |
| **Patient outcomes and workflow cohort** | Preintervention | 189 | 46 (24.3%) | DAWN protocol criteria^a^ | 7 (15.2%) |
|  |  |  |  | Died of cancer within 3 months | 4 (8.7%) |
|  |  |  |  | Pre-mRS >1 | 21 (45.7%) |
|  |  |  |  | EVT >1 during same hospital stay | 10 (21.7%) |
|  |  |  |  | EVT procedure >240 min^b^ | 7 (15.2%) |
|  | Postintervention | 86 | 21 (24.4%) | DAWN protocol criteria^a^ | 0 (0%) |
|  |  |  |  | Died of cancer within 3 months | 3 (14.3%) |
|  |  |  |  | Pre-mRS >1 | 14 (66.7%) |
|  |  |  |  | EVT >1 during same hospital stay | 0 (0%) |
|  |  |  |  | EVT procedure >240 min | 4 (19%) |
| **Anaesthetic Management Cohort** | Preintervention | 189 | 39 (20.6%) | Missing haemodynamic data/procedural time in AEMR | 32 (82.1%) |
|  |  |  |  | EVT procedure >240 min | 7 (17.9%) |
|  | Postintervention | 86 | 13 (15.1%) | Missing haemodynamic data/procedural time in AEMR | 9 (69.2%) |
|  |  |  |  | EVT procedure >240 min | 4 (30.8%) |

Abbreviations: mRS: modified Rankin scale; EVT: endovascular thrombectomy; AEMR: anaesthesia electronic medical record

^a^Selected for EVT based on DAWN protocol criteria; patients typically present 6 to 24 hours after symptom onset and are selected based on a clinical–imaging mismatch using advanced imaging.

^b^ 3 patients combined EVT procedure >240 min with other exclusion criteria.

**Supplemental Table 3.** Sample size variations by variable due to data availability

| Anaesthetic management cohort | Preintervention n | Postintervention  n |
| --- | --- | --- |
| Total sample size | 150 | 73 |
| Interhospital transport/inhospital stroke onset | 150 | 69 |
| Atrial Fibrillation | 149 | 73 |
| Diabetes mellitus | 149 | 73 |
| Hypertension | 149 | 73 |
| Previous myocardial infarction | 149 | 73 |
| Previous cerebral infarction | 149 | 73 |
| Initial NIHSS | 149 | 73 |
| Initial mRS | 145 | 69 |
| Initial ASPECT score | 147 | 67 |
| Bridging thrombolysis | 150 | 67 |
| Workflow process and patient outcomes cohort | Preintervention n | Postintervention  n |
| Total sample size | 143 | 65 |
| Initial mRS | 141 | 61 |
| Initial ASPECT score | 141 | 59 |
| Bridging thrombolysis | 143 | 59 |
| Invasive arterial blood pressure monitoring | 140 | 65 |
| Onset- Angio suite arrival | 100 | 49 |
| Onset-reperfusion | 102 | 42 |
| Door- Angio suite arrival | 125 | 48 |
| Door-reperfusion | 120 | 42 |
| Angio suite arrival-groin puncture | 128 | 63 |
| Groin puncture-reperfusion | 127 | 58 |
| TICI score 2b-3 | 143 | 65 |
| Interventional complications | 138 | 64 |
| ICH | 143 | 64 |
| Pre-mRS | 141 | 61 |
| mRS 0-1 | 141 | 61 |
| mRS 0-2 | 141 | 61 |
| mRS 5-6 | 141 | 61 |
| NIHSS post-EVT | 142 | 65 |
| NIHSS difference at 24 h | 142 | 65 |
| NIHSS at discharge | 131 | 58 |
| NIHSS difference at discharge | 131 | 58 |

Abbreviations: mRS: modified Rankin scale; ASPECT: Alberta stroke program early CT score; TICI: thrombolysis in cerebral infarction; ICH: intracerebral haemorrhage; NIHSS: national institutes of health stroke scale/score.
